# Evaluating the Tobii Pro Glasses 2 and 3 in static and dynamic conditions

**DOI:** 10.3758/s13428-023-02173-7

**Published:** 2023-08-07

**Authors:** V. Onkhar, D. Dodou, J. C. F. de Winter

**Affiliations:** 1 Department of Cognitive Robotics, Delft University of Technology, Delft, The Netherlands; 2 Department of Biomechanical Engineering, Delft University of Technology, Delft, The Netherlands

**Keywords:** Eye-tracking, Dynamic tasks, Eye-tracker accuracy, Computer vision

## Abstract

Over the past few decades, there have been significant developments in eye-tracking technology, particularly in the domain of mobile, head-mounted devices. Nevertheless, questions remain regarding the accuracy of these eye-trackers during static and dynamic tasks. In light of this, we evaluated the performance of two widely used devices: Tobii Pro Glasses 2 and Tobii Pro Glasses 3. A total of 36 participants engaged in tasks under three dynamicity conditions. In the “seated with a chinrest” trial, only the eyes could be moved; in the “seated without a chinrest” trial, both the head and the eyes were free to move; and during the walking trial, participants walked along a straight path. During the seated trials, participants’ gaze was directed towards dots on a wall by means of audio instructions, whereas in the walking trial, participants maintained their gaze on a bullseye while walking towards it. Eye-tracker accuracy was determined using computer vision techniques to identify the target within the scene camera image. The findings showed that Tobii 3 outperformed Tobii 2 in terms of accuracy during the walking trials. Moreover, the results suggest that employing a chinrest in the case of head-mounted eye-trackers is counterproductive, as it necessitates larger eye eccentricities for target fixation, thereby compromising accuracy compared to not using a chinrest, which allows for head movement. Lastly, it was found that participants who reported higher workload demonstrated poorer eye-tracking accuracy. The current findings may be useful in the design of experiments that involve head-mounted eye-trackers.

## Introduction

Eye-tracking, though seemingly a modern technique, is in fact by no means new, having been around in various forms for over 100 years (Płużyczka, 2018). As with any technology, its design and performance have improved over the decades, from invasive rods placed on users’ corneas and connected to sound-producing drums (Lamare, 1892) to noninvasive, remote screen-based systems as well as lightweight glasses equipped with infrared cameras. These contemporary eye-tracking devices are used in a variety of research fields, including psychology, marketing, art, sports, and human–computer interaction to investigate visual attention, cognitive processes, and user experience (Kredel et al., 2017; Meißner et al., 2019; Rosenberg & Klein, 2015).

However, with regard to the accuracy of eye-trackers, a mismatch has repeatedly been noted between their observed values and those reported in manufacturer specifications (Ehinger et al., 2019; Holmqvist, 2017; Morgante et al., 2012; Stuart et al., 2016). Therefore, there is a need to determine the accuracy of eye-trackers used in human subject research.

A variety of previous studies have evaluated the accuracy of eye-tracking technology. For example, Serchi et al. (2014) evaluated the Tobii TX300 remote (i.e., screen-based) eye-tracker. Their experiment involved four participants looking at a white dot that appeared sequentially for 2 seconds each in a grid of 13 dots, while standing at different distances or walking on a treadmill at a speed of 0.6 m/s or 1.1 m/s. They reported that the distance between the participant and the eye-tracker cameras was a critical factor in determining accuracy, but whether the participant was walking or not had little influence.

Recognizing the need to benchmark affordable remote eye-tracker models, Gibaldi et al. (2017) evaluated the Tobii EyeX, a low-cost eye-tracker attached to a screen. In their experiment, 15 participants were seated 0.7 m from the screen with a chinrest, and looked at circular targets appearing for 2 seconds on a grid, in random order. Their results showed that accuracy decreased with target eccentricity.

In a large-scale effort, Holmqvist (2017) assessed 12 eye-trackers with up to 194 participants per eye-tracker. They also analyzed participant characteristics that could potentially impact data quality, including eye color, eye makeup, pupil size, screen position, and the use of glasses. An unexpected result was that, compared to earlier studies of the same research group (Lång et al., 2011; Nyström et al., 2013), accuracy was worse. Several possible explanations were provided, including the low luminance of the environment, inexperienced or unmotivated experimenters, and the wide variety of participants. Generally, accuracy was found to be poorer for larger target eccentricities and for certain participant characteristics, such as blue eyes (in infrared, a blue iris appears darker than a brown iris), the use of makeup (mascara can be mistaken by the eye-tracker for a pupil because they both appear dark), glasses with antireflective coating, scratches, or dirt, and soft contact lenses (which may generate infrared reflections).

Concerning mobile, also known as head-mounted or wearable, eye-trackers, Stuart et al. (2016) assessed the accuracy of a Dikablis model, developed by Ergoneers. Thirty-four older participants (14 with Parkinson’s disease, 20 without) gazed at two targets placed 5°, 10°, and 15° apart in time with a metronome of 1 Hz for 20 seconds while seated and using a chinrest, standing and not moving their head, or walking on a treadmill. Accuracy was defined as the bias of saccade amplitude, with bias, in turn, defined as the difference between known target distance, i.e., eccentricity, and median saccade amplitude. It was evident that accuracy was poor and depended on the target eccentricity, but it did not seem to be significantly affected by whether participants sat, stood, or walked. The authors noted that the accuracy observed in the study was considerably worse than the 0.5° accuracy claimed by the manufacturer. They also observed that accuracy was better among participants with no visual correction as compared to those with correction.

Niehorster et al. (2020) conducted an experiment to investigate how accurately four wearable eye-trackers (Tobii Pro Glasses 2, SMI Eye Tracking Glasses 2.0, Pupil Labs Pupil in 3D mode, and Pupil Labs Pupil with Grip gaze estimation algorithm) recorded gaze when the glasses slipped on participants’ noses. Nine participants looked at (the center of) a grid containing eight ArUco markers at a distance of 1.5 m, while pronouncing vowels, making facial expressions, or moving the eye-trackers on their face using their hands. The authors observed that while the gaze estimates of the Tobii and Grip remained stable, the other two systems exhibited significant increases in gaze deviation when performing such movements, which raises concerns that they may not be suitable for use in dynamic scenarios.

Pastel et al. (2021) assessed the accuracy of the Eye Tracking Glasses 2.0 (SMI, Germany). Twenty-one participants were seated in front of a computer screen, used a chinrest, and sequentially performed three tasks: looking at stationary targets appearing at four locations, tracking a target moving in the shape of an infinity loop, and looking straight ahead at stationary targets at different distances. In line with previous studies, accuracy was found to be poorer for more eccentric gaze targets.

Finally, Hooge et al. (2022) compared six different eye-trackers (Pupil Core 3D, Pupil Invisible, SMI Eye Tracking Glasses 2 60 Hz, SeeTrue, Tobii Pro Glasses 2, Tobii Pro Glasses 3) in various conditions (e.g., standing still, walking along a circle, jumping). The results of four participants showed that the best accuracy occurred for the standing-still condition, but substantially poorer accuracy was obtained for walking, skipping, and jumping.

To summarize, a number of studies on remote and mobile eye-trackers have shown that eye-trackers are less accurate for targets at greater eccentricities (Gibaldi et al., 2017; MacInnes et al., 2018; Niehorster et al., 2020; Pastel et al., 2021; Stuart et al., 2016). There is less consensus on the effect of dynamic tasks, with earlier research (Serchi et al., 2014; Stuart et al., 2016) reporting no large differences between sitting, standing, and walking, while the recent study by Hooge et al. (2022) showed a clear reduction in accuracy with increased dynamicity from standing still to walking, skipping, and jumping. It should be noted, however, that Serchi et al. (2014) used a remote eye-tracker, which is normally not used while walking. Another factor to take into consideration in assessing the accuracy of mobile eye-trackers concerns the automated localization of the visual target in the camera image. This has been done by mapping the camera image to a reference image with the help of feature matching (MacInnes et al., 2018) or ArUco markers (Ehinger et al., 2019; Niehorster et al., 2020), or alternatively, by identifying the colored fixation target in the camera image (Hooge et al., 2022). These methods may introduce errors, depending on the method used. These challenges highlight the need for further research on the accuracy of mobile eye-trackers in dynamic tasks using appropriate computer-vision algorithms.

The current study investigates accuracy as a function of dynamicity by using two popular mobile eye-trackers: the Tobii Pro Glasses 2 and 3. The Tobii 2 is a widely used eye-tracker that has four eye cameras (2 per eye) and 12 illuminators (6 per eye), which are integrated into the frame of the glasses below and above the eyes. The Tobii 3 is a newer model with a more streamlined appearance resembling conventional glasses. It uses four eye cameras (2 per eye) and 16 illuminators (8 per eye) that are integrated into the lenses instead, for better positioning and supposedly more robust eye-tracking.

In our study, we assessed the performance of Tobii Pro Glasses 2 and 3 under three distinct conditions: the first encompassing only eye movements, the second incorporating both head and eye movements, and the third involving a combination of body, head, and eye movements. Investigating these three conditions allows for a comprehensive understanding of the devices’ performance under various realistic scenarios. Notably, our second condition represents an important (and until now, missing) bridge between seated, static trials and walking ones, two of the most commonly tested scenarios in research evaluating eye-trackers. We hypothesized that with each added layer of dynamicity, eye-tracking accuracy would worsen.

In addition to assessing eye-tracker accuracy for different task conditions, we evaluated how participant characteristics correlated with eye-tracker accuracy. We expected accuracy to be worse for participants who wore contact lenses and for participants with blue eyes because of their reduced contrast against pupils in infrared light (Holmqvist, 2017). Gender was not expected to be of influence (Holmqvist et al., 2022). Previous studies propose that eye-tracker accuracy might be affected by lighting conditions, considering that pupil diameter tends to vary in response to light (Hooge et al., 2021; Wyatt, 2010). According to documentation from Tobii, the accuracy of the Tobii 2 and Tobii 3 eye-tracking devices may be substantially compromised in environments with minimal lighting (1 lux) (Tobii, 2017b, 2022b). In the present study, although the lighting conditions were not as low, an investigation was conducted to determine the relationship between the recorded pupil diameter of the participants and the eye-tracker’s accuracy. Finally, we used the NASA Task Load Index (TLX) questionnaire to understand the association between eye-tracker accuracy and facets of perceived workload (i.e., mental demand, physical demand, temporal demand, performance, effort, and frustration), thus making it possible to assess whether eye-tracker accuracy is purely software- and hardware-related or also tied to participant state.

## Methods

### Participants

Thirty-six participants (20 male, 16 female) between the ages of 21 and 38 years (mean: 27.19, *SD*: 3.07, median: 26 years) were recruited via social media and direct contact to take part in the experiment between December 13, 2021 and February 4, 2022. Most were PhD candidates (22 participants) or employees at the Delft University of Technology or elsewhere (8 participants). The remaining participants were a postdoctoral researcher (1 participant) and (former) students (5 participants). Only people with normal visual acuity, corrected-to-normal vision using contact lenses, or low refractive errors (such that prescription lenses were not required for daily life activities) were eligible to participate.

Precautions were also taken against the spread of COVID-19 (sanitization of participants’ and experimenter’s hands, surfaces, and equipment touched, and social distancing whenever possible). All individuals provided written informed consent. The research was approved by the Human Research Ethics Committee of the Delft University of Technology (reference number 1832).

### Eye-trackers

Two head-mounted eye-trackers, the Tobii Pro Glasses 2 (firmware version 1.25.6-citronkola-0, head unit version 0.0.62) and the Tobii Pro Glasses 3 (firmware version 1.23.1+pumpa), were used to track participants’ gaze at 50 Hz and 100 Hz, respectively, and a forward-facing scene camera in each recorded their field of view at 25 frames per second and a resolution of 1920×1080 pixels. Note that the Tobii 2 allows for 100 Hz recordings by alternating the measurements from each eye (Holmqvist et al., 2022; Niehorster et al., 2020), an approach not taken in the present study. The Tobii 2 was used without its detachable protective lenses.

### Experimental setup

The experiment was conducted indoors in a workplace setting, with the seated trials performed in a private office and the walking trials in a nearby corridor, both of which were illuminated by natural and overhead lighting.

#### Seated trials

A pattern consisting of nine green dots was printed on white A1-size paper and attached on a wall; this arrangement included a central dot surrounded by eight equidistant peripheral dots, forming a circle with a diameter of 536 mm. Note that it was decided to use printed gaze targets instead of a digital display, such as a television screen, due to its portability and ease of setup, making it simpler to replicate the study.

A table of 1 m lateral width was placed against the wall, and a chinrest was clamped on the table’s opposite side, at its longitudinal center. At this distance from the wall, each dot had an eccentricity of 15° from the center. The dots themselves had a visual span of approximately 1.1° (20 mm diameter). The selection of a 15° eccentricity was informed by considerations of user comfort and applicability to real-life tasks. In tasks involving target detection, humans typically employ eye movements for small target eccentricities, and incorporate head movements for larger eccentricities to reduce eye strain (Stahl, 1999). Stahl found a mean eyes-only range across participants to be 35.8° (median of 25.3°), which is consistent with our 30° range and the ranges used in tests by eye-tracker manufacturers (Tobii, 2017b, 2022b). These assumptions align with eye movements during naturalistic tasks, such as walking, where individuals tend to focus on targets by employing a combination of eye and head movements. Standard deviations of eyes-in-head angles typically range from 5° to 10°, depending on factors such as terrain roughness (Bahill et al., 1975; Foulsham et al., 2011; Franchak et al., 2021; ‘t Hart & Einhäuser, 2012).

**Fig. 1:**
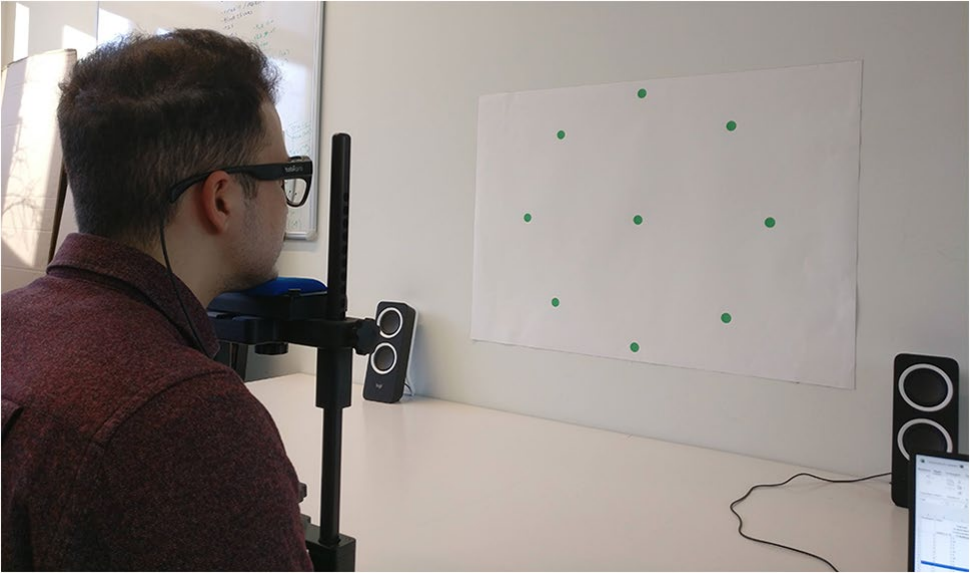
Seated trial with chinrest

**Fig. 2:**
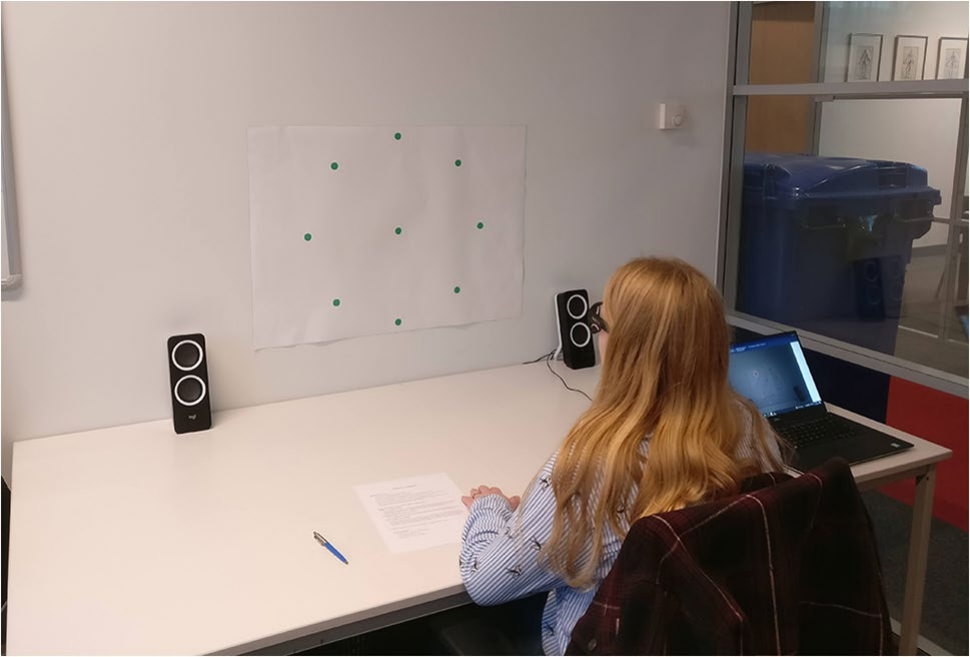
Seated trial without chinrest

The height of the chinrest was set prior to the experiment so that the central dot of the pattern was aligned with the experimenter’s eye level while seated in an office chair and using the chinrest. The height of the chinrest was not to be adjusted during the experiment, but participants were free to adjust the chair height to sit comfortably. The chinrest was unclamped and re-clamped to the table between trials, without compromising its preset height and position along the table. The chinrest was used without its removable forehead attachment, since it was not possible to press one’s forehead against it without having the eye-tracker collide with the setup. Two speakers were used in the seated trials to play audio instructions to guide participants’ gaze across the pattern. Figures 1 and 2 illustrate the seated trials with and without a chinrest, respectively.

#### Walking trials

**Fig. 3:**
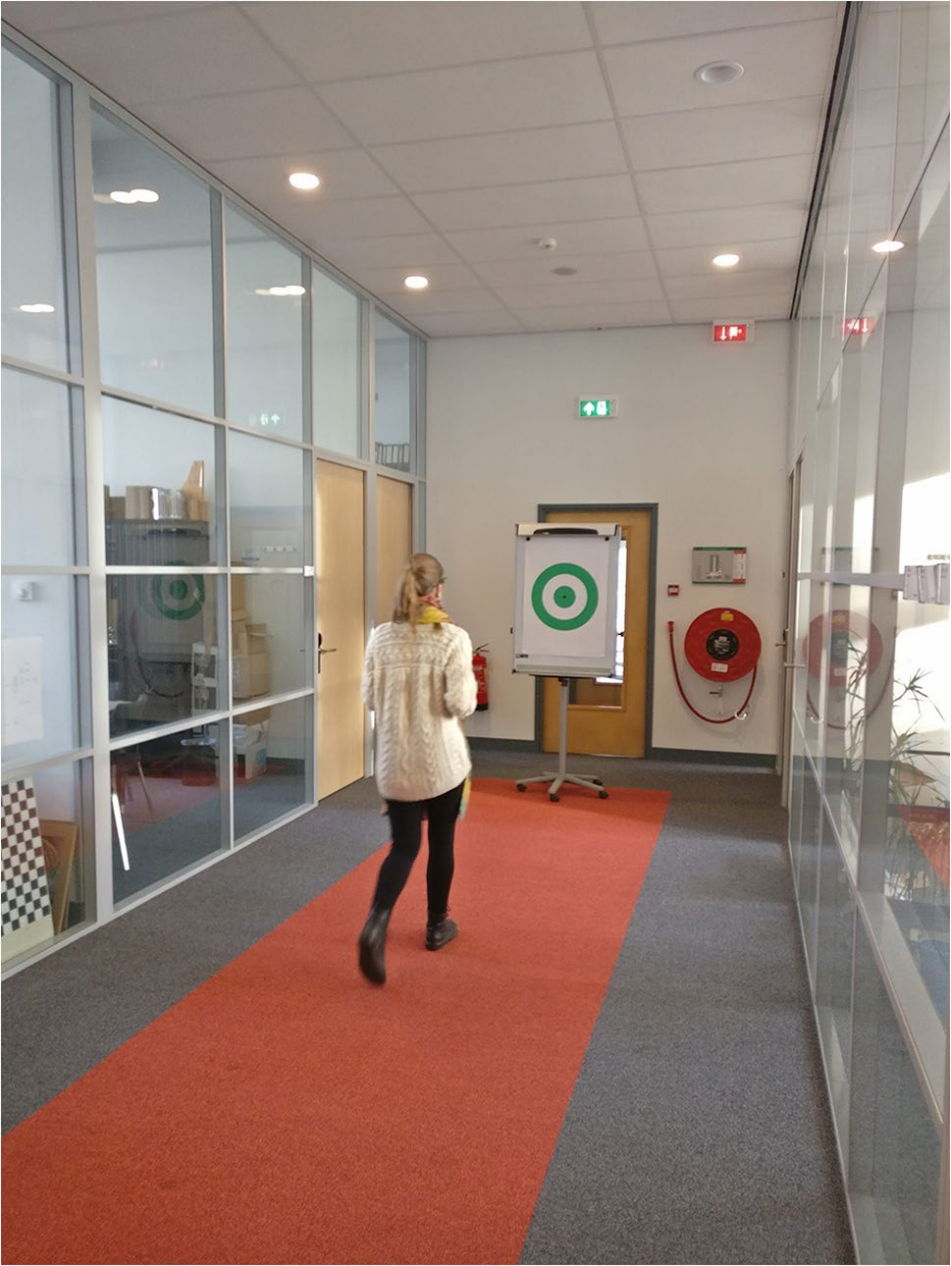
Walking trial

A green bullseye (480 mm outer diameter) was printed on A1-size paper and mounted on a mobile whiteboard. The whiteboard was placed at one end of a corridor, on the edge of a 21.7-m-long carpet. Participants would stand at the opposite end of the corridor before they commenced walking towards the bullseye during the trial. Figure 3 shows a walking trial in progress.

### Experimental design and procedure

The experiment involved three types of trials for each of the two eye-trackers, resulting in a total of six trials as shown in Table 1. A blocked design was implemented, with each block using one eye-tracker. Half of the participants, specifically those with odd participant numbers, began with a block using the Tobii 2, followed by a block using the Tobii 3. The remaining participants with even participant numbers started with a block using the Tobii 3 and then moved on to a block using the Tobii 2. Within each block, the sequence of the three dynamicity conditions was random.

**Table 1:** Experimental trials

Dynamicity condition	Eye-tracker	Permitted movements	Gaze target
Seated, with chinrest	Tobii 2	Eyes	Pattern of 9 green dots
Seated, without chinrest	Tobii 2	Eyes, head	Pattern of 9 green dots
Walking	Tobii 2	Eyes, head, body	Green bullseye
Seated, with chinrest	Tobii 3	Eyes	Pattern of 9 green dots
Seated, without chinrest	Tobii 3	Eyes, head	Pattern of 9 green dots
Walking	Tobii 3	Eyes, head, body	Green bullseye

Upon arrival, participants sanitized their hands, signed the consent form, and completed a questionnaire on their demographic data, eye color, and visual acuity. They were then briefed about the aim, procedure, and tasks of the experiment. Next, they put on one of the eye-trackers (depending on the predefined random order of trials assigned to them) and, if necessary, the eye-tracker nose pad size was adjusted for better comfort.

Participants’ gaze was then calibrated using a bullseye card that the participant held at arm’s length. A successful calibration was achieved when the participant’s gaze marker sufficiently overlapped with the bullseye for a specified period of time, criteria that were internally determined by the manufacturer’s software. All participants achieved successful calibration, and no participants were excluded because of failing to calibrate.

The robustness of the calibration was verified by asking the participant to move the card to multiple points of varying eccentricity (up, down, left, right), during which they looked at the card without rotating their head, using only their eyes. Recalibration was performed if there was insufficient overlap between the participant’s gaze marker and the bullseye. After successful verification, participants were not permitted to adjust the eye-tracker’s position on their faces until the upcoming trial was completed. Calibration and verification were performed before each trial, with participants standing in a designated area of the private office. Breaks were provided between trials if necessary.

In the seated trials with a chinrest, participants adjusted the chair height to sit comfortably, carefully placed their chin on the chinrest, and gazed at specific dots in the pattern (using only eye movements) for 12-second intervals each, following audio instructions in a synthesized female voice played in random order. The 12-second interval was chosen to ensure 10 seconds of available data per instructed dot (assuming it took participants no more than 2 seconds to respond to an instruction and focus on a new dot). The instructions were directions, each corresponding to a specific dot: “center,” “top,” “bottom,” “left,” “right,” “top left,” “top right,” “bottom left,” and “bottom right.” The central dot was called out, and hence to be visited, three times (at the start, middle, and end of the trial), and the remaining dots were called out twice each (in random order), in a trial that lasted just under 4 minutes.

Similarly, in the seated trials without a chinrest, participants gazed at specific dots in the pattern for 12-second intervals each, as per the audio instructions. They were also instructed in advance by the experimenter to look at the dots as they might naturally do, i.e., they were made aware they had the freedom to rotate their head as well as their eyes. The chinrest was unclamped and placed aside beforehand. These trials also lasted approximately 4 minutes and involved three visits to the central dot (at the start, middle, and end of the trial) and two visits each to the remaining dots in random order.

In the walking trials, when the corridor was free of passers-by and disturbances, participants walked from one of its ends to the bullseye at the other end, while keeping their gaze fixed on the center of the target. They were asked to walk normally, which meant eye, head, and body movements were all permissible. When they had reached a close distance to the bullseye, participants turned around, walked back to their starting position, and repeated this exercise once more. These trials lasted approximately 1 minute.

After each block of trials, i.e., after completing the three types of trials with a specific eye-tracker, participants completed the NASA TLX questionnaire, which polled six facets of workload: mental demand, physical demand, temporal demand, performance, effort, and frustration. The responses were recorded on a horizontal scale with 21 ticks, with anchors at the ends representing *very low* and *very high*, respectively. For the performance item, the anchors used were *perfect* and *failure*, respectively. Participants then put on the other eye-tracker and repeated the entire procedure once more, at which point the experiment was finished, and they were free to leave. Before the arrival of the next participant, the experimenter disinfected all surfaces and equipment that came into physical contact using alcohol wipes.

### Data preprocessing

Once the experiment was completed by all participants, the raw eye-tracking data were exported as separate .xlsx files. In addition, .mp4 video files from the Tobii project folders were used. The analysis used the variables “Gaze Point X” and “Gaze Point Y,” which represent the coordinates of the averaged gaze points for the left and right eyes in pixels, in the horizontal and vertical directions, respectively.

In the assessment of eye-tracker accuracy, it is important to focus on relevant accuracy indicators, rather than high-frequency jitter and blinks, which are commonly addressed in standard practice. Therefore, the data were filtered and blinks were removed. Specifically, the *x*- and *y*-data were passed through a moving median filter (e.g., De Winter et al., 2022; Jarodzka et al., 2012; Onkhar et al., 2021). The median filter had a 0.30-second interval and omitted missing data, i.e., any window containing missing values is the median of all non-missing elements in that window. A median filter removes noise and outliers while preserving edge information. That is, a median filter preserves fast movements like saccades, as opposed to smoothing filters, which would cause blurring of these rapid transitions.

**Fig. 4:**
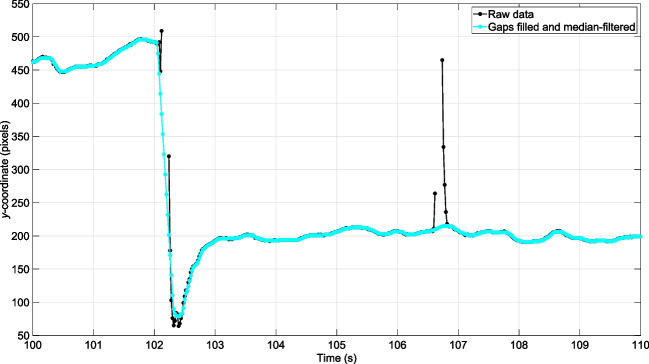
Filtering of the eye-tracking data for the Tobii 2. It can be seen that a blink at approximately 106.5 s was filled with data. Note that a *y*-coordinate of 1080 px corresponds to the bottom of the image (see also Fig. 8)

Figure 4 illustrates the filtering applied. Finally, the mean *x*- and *y*-positions were computed per video frame (i.e., two measurements per frame for the Tobii 2 and four measurements per frame for the Tobii 3).

The target coordinates were automatically extracted from the recorded video frames of the scene camera. For the seated trials, an image filter was applied so that the green dots stood out more clearly from the background. Next, MATLAB’s *imfindcircles* function (Yuen et al., 1990) was used to extract the nine dots (see Figs. 1 and 2). Various heuristics with regard to the expected distances between the dots were applied, to ensure that the dots were appropriately labeled (e.g., “center,” “top,” “top right”). For the walking trials, lines were fitted to the edges of the red carpet (see Fig. 3), and the intersection of the lines was used to estimate the approximate position of the bullseye. Next, the *imfindcircles* function was applied to estimate the coordinates of the bullseye in the scene camera image. Finally, a median filter was applied to the estimated coordinates of the target, using a time interval of five video frames (0.20 s) to remove possible jitter.

**Fig. 5:**
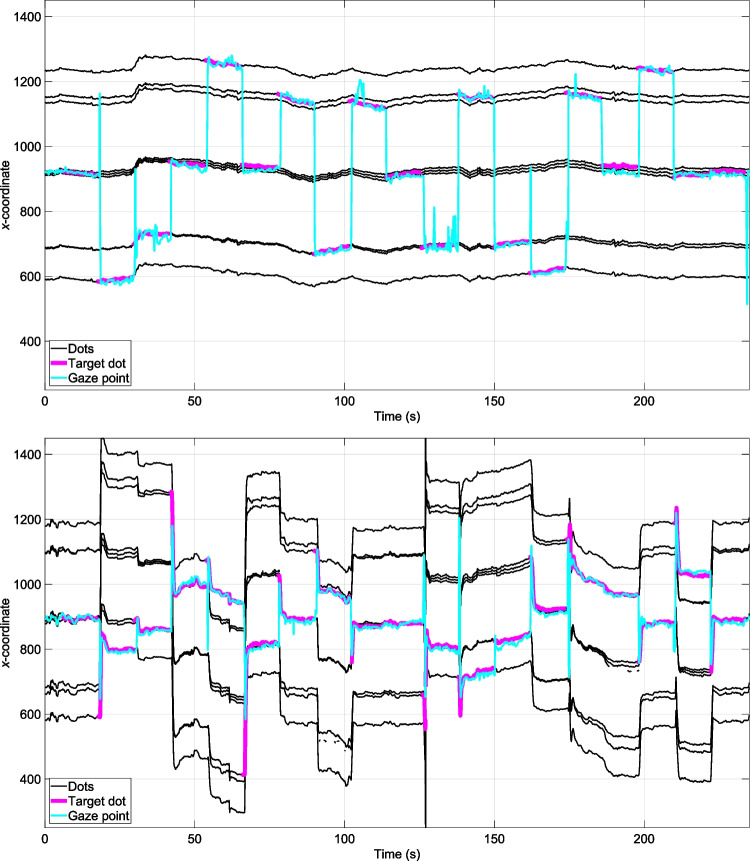
*x-*coordinate of the nine dots (in black) extracted using computer-vision, the instructed target dot highlighted in magenta, and a participant’s gaze (in cyan), in the seated trials with the Tobii 2, with chinrest (top figure) and without chinrest (bottom figure)

Figure 5 illustrates the type of data collected in a seated trial with chinrest (top figure) and without chinrest (bottom figure), for a participant wearing the Tobii 2. The figure shows the continuously tracked coordinates of the nine dots, as well as the target dot (the audio instruction onsets were automatically extracted from the audio recorded by the eye-tracker), and the gaze *x*-coordinate. It can be seen that the participant tracked the target dot accurately, and in the condition without chinrest, also rotated their head, as indicated by the changes in the position of the dots (especially after being instructed regarding a new target dot).

**Fig. 6:**
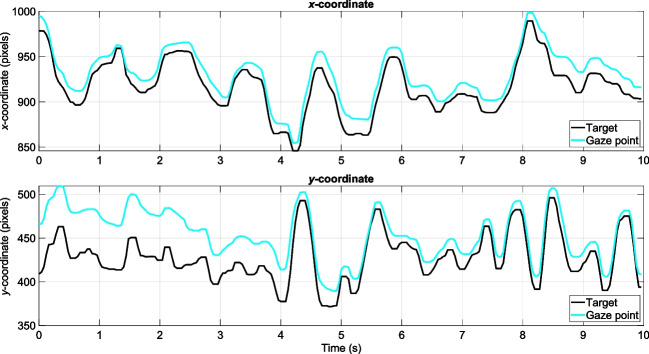
*x-*and *y-*coordinates of the estimated bullseye (in black) and filtered gaze (in cyan) for one participant in the walking trial (Tobii 3). The oscillating motion is caused by the participant’s gait

Figure 6 shows the equivalent target and gaze data for a walking trial. In the walking trials, the bullseye was not steady in the scene camera image, but oscillated according to the gait of the participant.

### Computation of angular distance with respect to target

For each video frame, the angular distance between Tobii’s instantaneous gaze point and the designated target was calculated. This was achieved by determining the angle between two vectors: the vector connecting the eyes to the gaze point, and the vector connecting the eyes to the target. The calculation was based on the dot product of the two vectors and the product of their magnitudes, as demonstrated in Eq. 1. The same approach has been previously employed in eye-tracking accuracy research (e.g., Aziz & Komogortsev, 2022, Eq. 4; Cercenelli et al., 2019, Eqs. 4 and 5; Mantiuk, 2017, Eq. 2; Xia et al., 2022, Eq. 26; and see Onkhar et al., 2021 using an equivalent formula using the cross-product and the dot-product).

1 $$\theta \left(i,j\right)=acos\;\left(\frac{{(x}_{i}-960)({x}_{j}-960)+{(y}_{i}-540){(y}_{j}-540)+{VD}^{2}}{\sqrt{{{(x}_{i}-960)}^{2}+{{(y}_{i}-540)}^{2}+{VD}^{2}}\cdot \sqrt{{{(x}_{j}-960)}^{2}+{{(y}_{j}-540)}^{2}+{VD}^{2}}}\right)$$

In Eq. 1, *x* and *y* are expressed in pixels, and (1,1) is the top-left corner of the image. *i* and *j* refer to the gaze coordinate and target coordinate at that moment, respectively. A constant is subtracted from the *x*- and *y*-coordinates to ensure that the angular distance is computed relative to the center of the scene camera image (e.g., Mantiuk, 2017). More specifically, for the *x*-coordinates, 960 pixels are subtracted, or half the screen width. For the *y*-coordinates, 540 pixels are subtracted, being half the screen height.

**Fig. 7:**
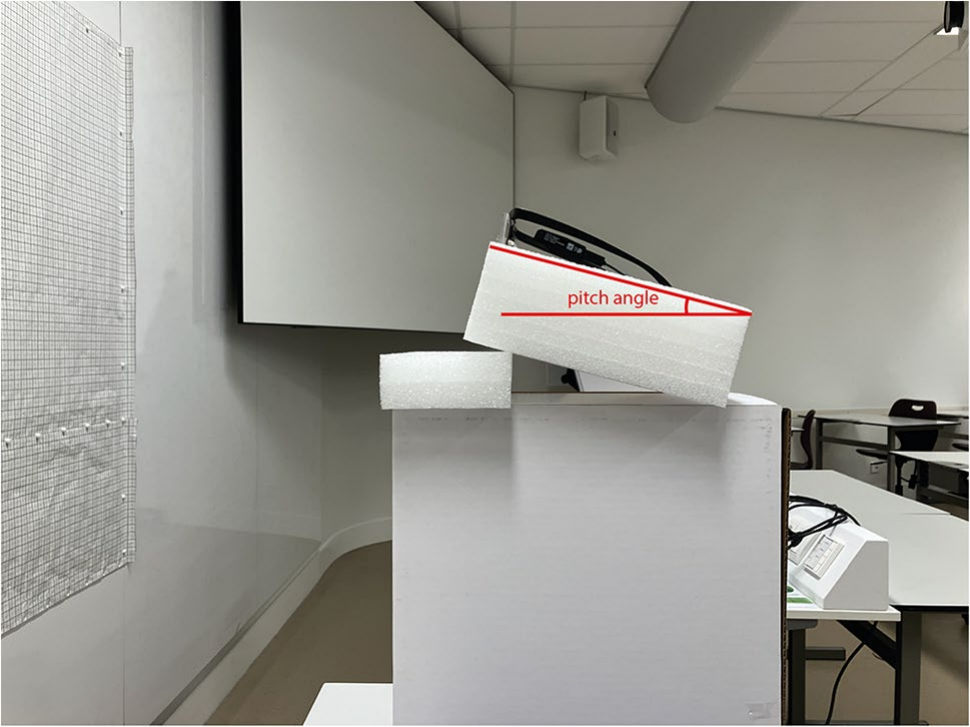
Setup for estimating the *VD* parameter. Graph paper consisting of 1×1 cm squares was stuck to the wall, and the Tobii was located at a distance of approximately 1 m from the wall

**Fig. 8:**
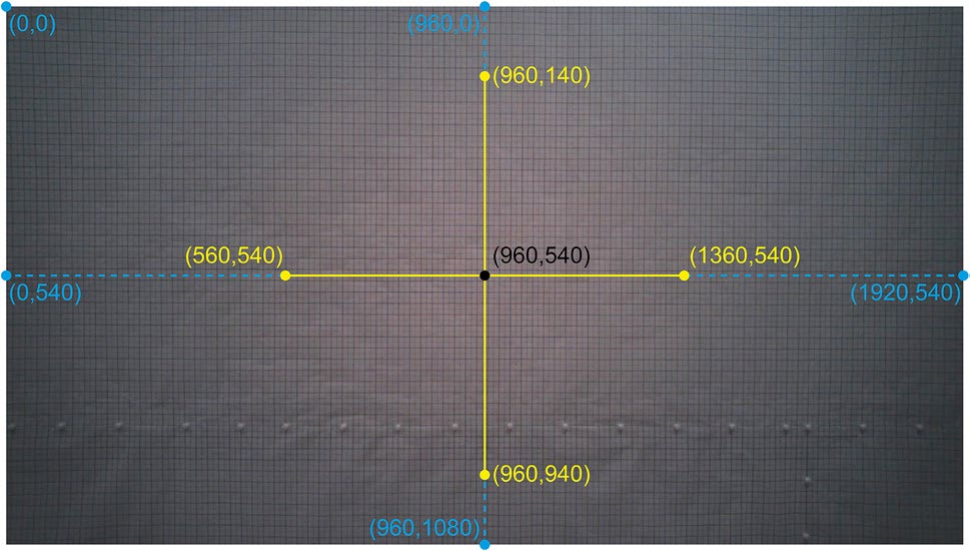
View of the scene camera pointing at the graph paper in the setup shown in Fig. 7. The coordinates used in the analysis are shown. The distance to the edges (blue) was used to estimate the camera field of view, and the distance to points at 400 px eccentricity (yellow) was used to estimate the viewing distance (*VD*)

Viewing distance (*VD*) is a constant that relates to the magnification factor of the scene camera. If placing the Tobii closer (or farther) from the wall, the same translation in pixels corresponds to a proportionally smaller (or larger) translation in millimeters. *VD* can be interpreted as the virtual viewing distance, that is, the ratio between the distance between the Tobii scene camera and the wall in mm and the pixel size in mm. The *VD* parameter was determined by placing the Tobii approximately 1 m away from a wall with graph paper on it (Figs. 7 and 8). The Tobii glasses were tilted so that the grid was vertically aligned with the borders of the camera image. Through manual and automated evaluations of the distances between grid lines, it was concluded that the camera view exhibited negligible distortion, aside from the outer few centimeters of the grid. Consequently, we opted to proceed without conducting a camera calibration designed to rectify such distortions. A screenshot of the camera view was made, and the distance in millimeters from the image center to various points (Fig. 8) was determined with the help of the grid. Using the mean distances reported in Table 2 for a 400-pixel (px) eccentricity, *VD* was estimated to be 1132.4 px and 912.8 px for the Tobii 2 and 3, respectively. In other words, although both eye-trackers offered the same image resolution of 1920×1080 px, the Tobii 3 offered a larger field of view.

**Table 2:** Measured distances between coordinates shown in Fig. 8 for the Tobii 2 and 3

Model	Distance between scene camera and wall (mm)	Pitch angle of Tobii (°)	Distance between edge coordinates and center (°)	Distance between center and coordinates at 400 px eccentricity (°)
Tobii 2	1010	12	(0, 540): 846 mm (40.0°) (1920, 540): 850 mm (40.1°) (960, 0): 475 mm (25.2°) (960, 1080): 481 mm (25.5°)	(560, 540): 357 mm (19.5°) (1360, 540): 358 mm (19.5°) (960, 140): 354 mm (19.3°) (960, 940): 358 mm (19.5°)
Tobii 3	890	1	(0, 540): 950 mm (46.9°) (1920, 540): 970 mm (47.5°) (960, 0): 524 mm (30.5°) (960, 1080): 529 mm (30.7°)	(560, 540): 386 mm (23.4°) (1360, 540): 394 mm (23.9°) (960, 140): 390 mm (23.7°) (960, 940): 390 mm (23.7°)

The distance between coordinates in mm was converted to degrees using *atan*(*d*/*D*), where *d* is the distance between the point and the image center in millimeters, and *D* is the distance between the scene camera and the wall in millimeters. For the Tobii 2, the horizontal and vertical fields of view (FOVs) were established to be 80.1° and 50.7°, which are close to the specifications reported by Tobii (82° and 52°; Tobii, 2017a). For the Tobii 3, the horizontal and vertical FOVs were estimated at 94.4° and 61.2°, again close to the manufacturer specifications (95° and 63°; Tobii, 2022a). The pitch angle was measured with a mobile app (RateFast, 2015)

In the seated trials, instructions to gaze at the dots were provided a total of 19 times (center dot: 3 times, other eight dots: 2 times each). The time interval between instructions to fixate on each dot was 12 seconds. Because the participant would need time to shift focus from one dot to the next (see Fig. 5), the first 2 seconds were discarded, and the angular distance was averaged over the remaining 10-second interval (i.e., 250 frames). For the walking trials, the participant walked up to the bullseye twice. Angular distance was averaged over a 10-second interval (see Fig. 6) for each walk. The intervals for the walking trials were automatically extracted, with their end point being approximately when the red carpet went out of the scene camera’s view and the starting point being 10 seconds before that moment.

The accuracy of the gaze of a participant in an experimental condition was calculated by computing the mean angular distance θ over the time intervals of that participant and condition. For the seated trials, 19 time intervals of 10 seconds each were available. For the walking trials, participants performed the task twice, and two time intervals of 10 seconds were used.

## Results

Of the 36 participants, two participants (1 male, 1 female) completed only the Tobii 2 trials of the experiment due to a malfunction in the Tobii 3. Four other participants completed the experiment in two sessions spread across two separate days for the same reason. Furthermore, for one participant, the results for one condition (Tobii 3, without chinrest) were not available because of an error by the experimenter in carrying out the trials in their predefined order. Finally, for one of the participants in the walking trial with the Tobii 3, one of the two 10-second intervals was declared invalid due to an individual walking in front of the bullseye; consequently, the results of this trial rely on the data gathered from only one of the two time intervals.

### Accuracy of the eye-trackers

First, we determined the accuracy of the Tobii 2 and 3, where accuracy refers to the mean angular distance from the target. Table 3 shows the accuracy, averaged across participants and all nine dots, for the six experimental conditions. According to a two-way repeated-measures analysis of variance (ANOVA), there was a significant effect of Tobii model, *F*(1,32) = 31.7, *p* < 0.001, partial η^2^ = 0.50, and of the level of dynamicity (i.e., with chinrest, without chinrest, or walking), *F*(2,64) = 5.25, *p* = 0.008, partial η^2^ = 0.14. There was also a significant Tobii model × dynamicity interaction, *F*(2,64) = 5.25, *p* = 0.008, partial η^2^ = 0.14.

**Table 3:** Accuracy per experimental condition (in degrees) for all nine dots and for the central dot only. The mean, standard deviation (*SD*), and median across participants are reported

		All dots			Only central dot			
Dynamicity condition	Eye-tracker	Mean	*SD*	Median	Mean	*SD*	Median	*n*
Seated, with chinrest	Tobii 2	2.77	1.49	2.40	1.44	1.90	1.04	36
Seated, without chinrest	Tobii 2	1.99	1.45	1.55	1.58	1.96	1.03	36
Walking	Tobii 2	3.53	2.70	2.86				36
Seated, with chinrest	Tobii 3	1.78	1.09	1.54	1.21	0.79	0.96	34
Seated, without chinrest	Tobii 3	1.60	0.98	1.27	1.23	0.96	0.86	33
Walking	Tobii 3	1.74	0.90	1.59				34

For the walking trials, there was only one dot (i.e., the bullseye)

Post hoc paired-samples *t*-tests showed that for the Tobii 2, the chinrest condition yielded significantly poorer accuracy than the without-chinrest condition (*p* = 0.001) but not compared to the walking condition (*p* = 0.123). Furthermore, the without-chinrest condition yielded significantly better accuracy than the walking condition (*p* = 0.003). On the other hand, for the Tobii 3, there was no significant difference between the chinrest condition and the without-chinrest condition (*p* = 0.134) or the walking condition (*p* = 0.859). Also, the without-chinrest condition yielded no significant difference from the walking condition (*p* = 0.404). Upon comparison of the two eye-trackers, it was observed that the Tobii 3 had significantly better accuracy than the Tobii 2 for the chinrest condition (*p* < 0.001) and walking condition (*p* < 0.001), but not for the without-chinrest condition (*p* = 0.051).

**Fig. 9:**
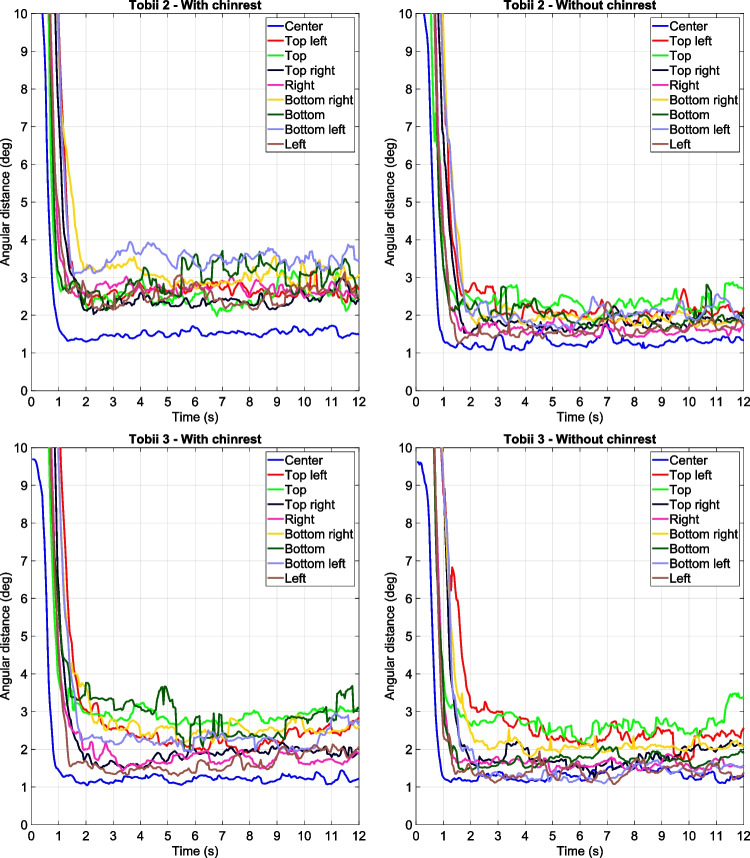
Angular distance to instructed dots, averaged across participants, for the Tobii 2 (top) and the Tobii 3 (bottom) and for the chinrest condition (left) and the without-chinrest condition (right). Note that the accuracy was determined for the last 10 seconds of the 12-second interval

Note that the accuracy of the eye-trackers in the seated trials, as presented above, was calculated by computing the average across all nine dots. Table 3 also shows the accuracy specifically for the center dot. It can be seen that the mean accuracy for the center dot is markedly better as compared to all dots. This difference in accuracy can also be seen in Fig. 9, which shows the angular distance to each instructed dot in the seated trials. It can be observed that for both eye-trackers, the center dot was detected more accurately than the others. In particular for the Tobii 2 with chinrest, the eccentric dots showed poor accuracy compared to the center dot

### Movement of the gaze target

**Fig. 10:**
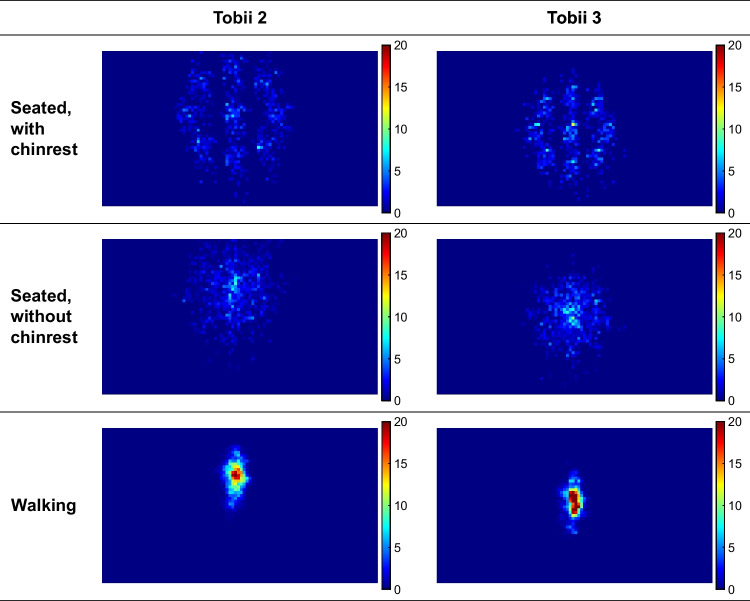
Heatmaps of the location of the targets (i.e., instructed dots for the seated trials, bullseye for the walking trials) across all 10-second intervals of all participants. The colormap represents the number of target points in the 20×20-pixel square, normalized so that the total of all squares equals 1000

Heatmaps were created to better understand the underlying causes of the relatively poor accuracy of the Tobii 2. The heatmaps, shown in Fig. 10, were created by dividing the camera image into squares of 20×20 pixels. It can be seen that the target was on average higher in the scene camera for the Tobii 2 compared to the Tobii 3. It can also be seen that participants without a chinrest were inclined to center the target dot in their field of view. That is, for the chinrest condition, participants looked at the dots by turning only their eyes, in accordance with the instructions. In contrast, during the without-chinrest condition, they also turned their heads, reducing the need to turn their eyes towards large eccentricities. Similarly, for the walking trials, in which there was only one target, participants tended to keep the target in the center of their view.

A restriction of movement was also associated with a larger amount of missing data. Specifically, the percentage of missing gaze data computed per video frame, and after filtering (see Methods) was 3.07, 1.88, and 0.12% for the chinrest, without-chinrest, and walking conditions of the Tobii 2, and 1.42, 0.58, and 0.12% for the Tobii 3, respectively. Before filtering, these values were 6.07, 3.96, and 1.41% for the Tobii 2, and 3.63, 2.43, and 1.38% for the Tobii 3.

Subsequent to the above observations, an analysis of target speed within the camera image was conducted. Table 4 shows the mean speed of the identified target, averaged across the 10-second measurement interval. The instantaneous speed was computed using Eq. 1 for *x*- and *y*-coordinates for successive video frames. The results presented in Table 4 corroborate the efficacy of the chinrest in limiting head motion in comparison to the other conditions. There was no detectable effect of the Tobii model, which can be explained by the fact that the speed of the target is solely caused by participant movement, not by the eye-tracker itself. Specifically, according to a two-way repeated-measures ANOVA, there was no significant effect of the Tobii model, *F*(1,32) = 0.09, *p* = 0.766, partial η^2^ = 0.00, but a strong effect of the level of dynamicity, *F*(2,64) = 215.9, *p* < 0.001, partial η^2^ = 0.87. There was also no significant Tobii model × dynamicity interaction, *F*(2,64) = 0.14, *p* = 0.873, partial η^2^ = 0.00.

**Table 4:** Mean speed of the target in the camera images per experimental condition (in degrees per second). The mean, standard deviation (*SD*), and median across participants are reported

Dynamicity condition	Eye-tracker	Mean	*SD*	Median	*n*
Seated, with chinrest	Tobii 2	0.28	0.08	0.27	36
Seated, without chinrest	Tobii 2	0.83	0.70	0.68	36
Walking	Tobii 2	8.57	3.04	7.99	36
Seated, with chinrest	Tobii 3	0.28	0.08	0.26	34
Seated, without chinrest	Tobii 3	0.81	0.47	0.67	33
Walking	Tobii 3	8.77	3.88	8.39	34

### Self-reported workload

To gain a deeper understanding of whether human workload experience is linked to eye-tracking accuracy, we examined the self-reported workload data from participants. Table 5 lists the perceived workloads when using the Tobii 2 and 3. It can be seen that the Tobii 3 resulted in statistically significantly lower physical demand, effort, and frustration compared to the Tobii 2.

**Table 5:** Mean and standard deviation of self-reported workload for Tobii 2 (*n* = 36) and Tobii 3 (*n* = 34). Also shown are the results of a paired-samples *t*-test

	Tobii 2		Tobii 3		
	Mean	*SD*	Mean	*SD*	*t*-test
TLX Mental demand (%)	29.7	22.8	27.9	19.9	*t*(33) = 1.17, *p* = 0.249
TLX Physical demand (%)	30.8	22.0	26.0	22.5	***t*(33) = 2.56, *p* = 0.015**
TLX Temporal demand (%)	18.5	17.1	19.0	18.4	*t*(33) = 0.19, *p* = 0.851
TLX Performance (%)	30.3	21.6	29.1	21.8	*t*(33) = 1.38, *p* = 0.176
TLX Effort (%)	39.4	26.2	32.2	21.2	***t*(33) = 2.78, *p* = 0.009**
TLX Frustration (%)	30.3	26.7	21.2	22.5	***t*(33) = 2.77, *p* = 0.009**

Scores were converted to a scale from 0% (minimum possible) to 100% (maximum possible). *p* < 0.05 is listed in boldface

### Individual differences

**Fig. 11:**
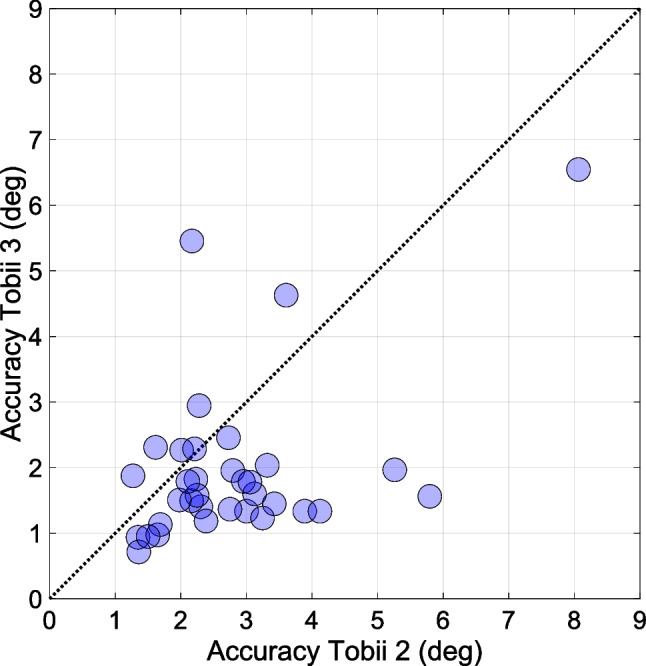
Overall eye-tracking accuracy (averaged across the three dynamicity conditions) for the Tobii 2 and 3. Each circular marker represents a participant (*n* = 34). The dotted line represents the line of equality

In order to better understand the underlying factors that contribute to variations in eye-tracking accuracy, we examined individual differences among participants using the devices. The Pearson correlation between participants’ overall accuracy (averaged across the dynamicity conditions) between the Tobii 2 and Tobii 3 was *r* = 0.56 (*p* < 0.001). This finding suggests that individual differences, such as unique eye characteristics or behavioral patterns, affected the performance of both eye-trackers; see Fig. 11 for the tracking accuracy for the Tobii 2 and 3 per participant.

Point-biserial correlations of participants’ overall accuracy (*z*-transformed and subsequently averaged over all six conditions) did not reveal a statistically significant association with participant gender, whether or not the participant was wearing contact lenses, whether the participant had a particular eye color, and the participant’s pupil diameter averaged across all conditions (see Table 6). However, there were significant associations with self-reported workload (physical demand, temporal demand, and frustration). These findings suggest that eye-tracking accuracy may be more influenced by participants’ workload rather than their physical characteristics, highlighting the importance of considering human factors in eye-tracking research and technology development.

**Table 6:** Means, sample sizes (*n*), and standard deviations of participants’ characteristics and self-reported workload, together with the Pearson product-moment correlation coefficient with overall angular distance (*n* = 36)

	Mean	*n*	*r*	*p*
Gender (0: female / 1: male)	0.56	16 / 20	-0.09	0.619
Contact lenses (0: no / 1: yes)	0.22	28 / 8	0.16	0.341
Brown eyes (0: no / 1: yes)	0.39	22 / 14	0.19	0.276
Blue eyes (0: no / 1: yes)	0.14	31 / 5	0.23	0.172
Other eye color (0: no / 1: yes)	0.47	19 / 17	-0.34	0.040
	Mean	*SD*	*r*	*p*
Pupil diameter (mm)	3.80	0.66	0.06	0.732
TLX Mental demand (%)	28.5	20.4	-0.13	0.456
TLX Physical demand (%)	28.3	21.1	0.41	**0.013**
TLX Temporal demand (%)	18.3	17.1	0.46	**0.005**
TLX Performance (%)	29.2	21.1	0.09	0.598
TLX Effort (%)	35.8	22.3	0.32	0.056
TLX Frustration (%)	25.9	22.8	0.34	**0.040**

Scores for the NASA TLX were averaged across the two eye-trackers (only the Tobii 2 in two participants) and converted to a scale from 0% (minimum possible) to 100% (maximum possible). *p* < 0.05 is listed in boldface. For binary variables (gender, contact lenses, eye color), the Pearson product-moment correlation coefficient is equivalent to the point-biserial correlation coefficient. Other eye colors include hazel, gray, green, and amber

**Fig. 12:**
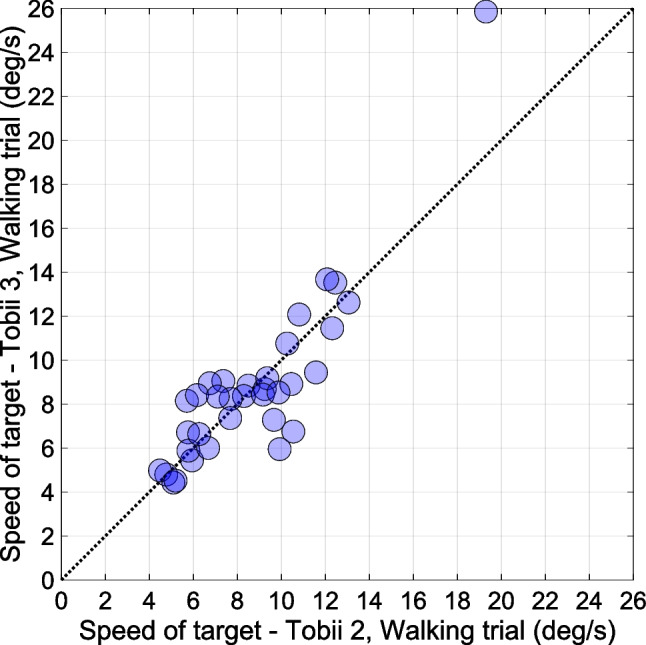
Mean speed of the target in the camera image in the walking trials for the Tobii 2 and the Tobii 3. Each circular marker represents a participant (*n* = 34). The dotted line represents the line of equality

Finally, we investigated head movement as a possible factor influencing eye-tracking accuracy. Figure 12 illustrates that the speed of movement of the target was strongly person-specific, with some participants having a more dynamic gait than others regardless of Tobii model (*r* = 0.88, *p* < 0.001). However, the speed of the target did not correlate significantly with eye-tracking accuracy (*r* = -0.26, *p* = 0.133, *n* = 36 for the walking trials of the Tobii 2; *r* = -0.12, *p* = 0.509, *n* = 34 for the walking trials of the Tobii 3). That is, participants’ eye-tracking accuracy was not significantly related to the speed of their head movement while walking.

## Discussion

In this study, the accuracy of Tobii Pro Glasses 2 and Tobii Pro Glasses 3 was compared under three distinct dynamicity conditions representing varying levels of freedom in movement: using a chinrest, without a chinrest, and while walking.

The accuracy of the Tobii 2 was found to be better without a chinrest, opposite to the standard approach for remote eye-trackers that recommends using a chinrest for enhanced accuracy (e.g., Holmqvist et al., 2011; Minakata & Beier, 2021; Niehorster et al., 2018). Interestingly, some researchers have used chinrests with mobile eye-trackers (Wang & Grossman, 2020; Werner et al., 2019). We initially hypothesized that eye-tracker accuracy would deteriorate with increasing levels of movement freedom; however, this did not turn out to be the case. The improved accuracy for the off-center dots without a chinrest can be ascribed to participants turning their heads, thereby reducing the need for gazing at large eccentricities. Eye-trackers generally exhibit better accuracy at smaller target eccentricities (MacInnes et al., 2018; Stuart et al., 2016), a notion supported by our seated trial results, wherein the central dot demonstrated the highest accuracy. Consequently, the use of a chinrest with a mobile eye-tracker is not recommended.

Our study also showed that the Tobii 3 exhibited statistically significant better accuracy than the Tobii 2 during the walking condition. This enhanced accuracy of the Tobii 3 may be attributed to the incorporation of extra illuminators and the more optimal placement of its eye-tracking cameras. Moreover, the Tobii 3’s design possibly renders it more resistant to inaccuracies resulting from vibrations and other perturbations. Additionally, the Tobii 2 is characterized by a downward pitch angle of its scene camera (as also noted by Niehorster et al., 2020; Rogers et al., 2018; Thibeault et al., 2019, and which can be seen in the heatmaps in Fig. 10). This deviation may compromise accuracy, as the central gaze point is situated at an eccentricity.

Another finding was that the difference in eye-tracking accuracy for the Tobii 3 during walking (averaging at 1.74°) and seated trials (averaging at 1.78° with chinrest and 1.60° without chinrest) was only small and not statistically significant. This result was unexpected, since research has suggested that eye-tracking accuracy is compromised during dynamic tasks such as walking (Hooge et al., 2022). In line with the above explanation for the chinrest vs. without-chinrest condition for the Tobii 2, a possible explanation is that participants were able to freely rotate their heads and bodies while walking, which helped to keep the target in the center of their field of view. This interpretation is supported by the heatmaps in Fig. 10, which show relatively centralized gaze patterns for the walking trials. Additionally, the results suggest that participants were able to keep their gaze on the target while walking, regardless of how much head movement the participant exhibited. The effectiveness with which participants are able to track a target while walking can be attributed to the vestibular-ocular reflex, i.e., the reflexive eye movement that helps to stabilize the gaze on a target while the head is moving (Dietrich & Wuehr, 2019; Moore et al., 1999). Future research could examine the factors that contribute to eye-tracking accuracy in dynamic tasks, including the role of head and body movements in maintaining a stable gaze. Additionally, it would be interesting to explore the generalizability of the findings to other mobile eye-trackers and different types of dynamic tasks, such as those requiring rapid head turns (e.g., crossing a road as a pedestrian, scanning a traffic scene as a driver) or tasks that require fast walking or running.

The present study examined the potential influence of several participant characteristics, namely gender, the use of contact lenses, eye color, and mean pupil diameter, on the overall accuracy of the eye-trackers. A sample of 36 participants was used, and the results did not reveal statistically significant associations with the accuracy of the eye-trackers. Previous research (Holmqvist, 2017; Nyström et al., 2013) suggested that contact lenses may reduce the accuracy of eye-trackers due to multiple corneal reflections. The results of our study did not support this hypothesis. It is possible that the Tobii eye-trackers were designed to accurately track eye movements even in the presence of these reflections. Future research could examine the effect of a larger variety of participant characteristics, including eye shape, and using larger sample sizes.

Our findings shown in Table 3 indicated that the mean accuracies across participants for the Tobii 2 and Tobii 3 in the chinrest condition were 2.77° and 1.78°, respectively. If selecting only the center target, the mean accuracies for the Tobii 2 and 3 are 1.44° and 1.21°, respectively. Our findings are in the ballpark of previous studies that have evaluated the Tobii 2, with mean accuracies between 1.19° and 5.25° for various target eccentricities (Niehorster et al., 2020). It is noteworthy, however, that even our accuracies for the center target alone are worse than those reported by Tobii, which reported a mean accuracy of 0.62° for the Tobii 2 in an unpublished test report (Tobii, 2017b), and 0.5° (for a central target) to 0.8° (for ‘common gaze angles’) in a Tobii 3 test report (Tobii, 2022b). There are several potential factors that could explain the discrepancies in accuracy observed between Tobii’s results and the current study:

- One factor is that Tobii (2017b) used another definition of accuracy. Following Holmqvist et al. (2011), we computed the angular distance per video frame, and subsequently calculated the mean angular distance, which is always a positive value, across those frames. Hence, in our case, accuracy represents the overall angular distance from the target. On the other hand, Tobii (2017b) and others (e.g., MacInnes et al., 2018) have defined accuracy as bias, a component of accuracy, which they calculated by determining the mean gaze point over a time interval and then computing the angular difference between this mean gaze point and the target. Bias, reflecting the average deviation from the true value, can present a misleadingly low value compared to the overall accuracy (absolute error), by allowing overestimations and underestimations to offset each other. Therefore, bias should always be interpreted together with precision (which has been defined in different ways in the literature). For completeness, we offer bias and precision values for our experimental conditions in Appendices Tables 7 and 8. Bias values can be seen to be indeed smaller than the accuracy values presented in Table 3.
- Furthermore, in our seated trials, 3 out of 19 trials concerned the central dot, whereas the remaining 16 involved a dot at 15° eccentricity. In contrast, Tobii AB employed a more evenly distributed range of eccentricity values, which therefore constituted a less demanding evaluation. Specifically, the Tobii 2 test report (Tobii, 2017b) investigated one central dot, four dots situated at approximately 7° eccentricity, and another four dots at 10° eccentricity. Similarly, the Tobii 3 report (Tobii, 2022b) evaluated five dots within a 5° range, four dots at approximately 8° eccentricity, and four additional dots at 14° eccentricity. The fairness of each test may be a matter of debate: Despite both our study and Tobii’s shared interest in targets at 15° eccentricity or below, our research was primarily concerned with targets at 15° eccentricity, the natural limit of eccentric gaze. In contrast, Tobii’s evaluation included only a limited number of targets in close proximity to 15° eccentricity.
- Another consideration is the extended gaze duration employed in our seated trials, wherein accuracy was calculated over 10-second intervals, which is considerably longer than Tobii’s 1-second window (Tobii, 2017b, 2022b). This decision was made to capture eye-tracker variability, although it introduced possible drawbacks, such as a challenge for participants to maintain focus. Figure 9 shows no evident difficulty in sustaining attention, as the mean angular distance across participants remained approximately constant throughout the measurement interval. Furthermore, it is important to note that large angular deviations should not necessarily be ascribed to participant inattention; eye-tracker inaccuracies themselves may also be a contributing factor.
- Another potential explanation for the better accuracy reported by Tobii (2017b) is that they may have included participants whose eyes were better trackable. Indeed, it should be noted that the median value of participants, as presented in Table 3, is lower than the mean value. This observation suggests that a small number of participants may be responsible for a disproportionately large portion of the observed inaccuracy. This point was also highlighted by Holmqvist (2017), who noted: “*we did our best to record all sorts of participants with troublesome features, while a typical study would try to exclude participants with glasses, mascara, squinting, unsuitable pupil sizes and other issues already during recruitment*” (p. 20).
- Also, based on correspondence received from Tobii AB in response to a preprint of this work, it was proposed that differences in firmware might have partially accounted for the observed discrepancy. In Tobii’s tests of the Tobii 2, firmware 1.16.1 was used (Tobii, 2017b), whereas our study used the latest version accessible to users, 1.25.6-citronkola-0. Likewise, for the Tobii 3, our experiment used the most recent firmware version available at the time, 1.23.1+pumpa, while Tobii’s report, published later that year, made use of 1.28.1-granskott (Tobii, 2022b).
- As a final point, the methods of filtering and data processing implemented could potentially have had an influence. We developed the filtering algorithm to adequately handle artifacts such as blinks (see Fig. 4), while also providing a robust estimate in the event of extensive missing data and jitter. In this sense, it should be noted that the filtering improves the apparent accuracy of the eye-tracker by an average of about 0.14° compared to using unfiltered data. In addition, it should be noted that during the seated trials with the Tobii 2, the top target occasionally disappeared from the camera view due to the previously mentioned tilt angle of the eye-tracker. This resulted in a gap in the measurement data, which in this paper is not considered as missing eye-tracking data or inaccurate measurement. The angular deviation also occasionally assumed  very high values, which has a relatively large influence on the accuracy. One possible way to address this would be to define accuracy not as the mean angular deviation, as we have done, but as the median angular deviation. When this is done, the accuracy improves by approximately 19% compared to the values in Table 3.

Our study found that Tobii 3 had more accurate eye-tracking abilities, and also yielded statistically significantly lower physical demand, effort, and frustration than the Tobii 2, something which may be due to its more ergonomic design. In support of this, the experimenter noted that some participants reported partial obstruction of their view by the corners of the Tobii 2 frame when settling into the chinrest ahead of those trials and gazing eccentrically at the “left” and “right” target dots. An accuracy–workload correlation was also found at the level of participants, with participants who had better eye-tracking accuracy experiencing lower physical demand, temporal demand, and frustration. A possible explanation for the latter correlations is that participants who were less motivated and more fatigued exhibited increased bodily movement and a decreased ability to keep their eyes on the target. It is also possible that participants who had experienced experimental nuisances, such as calibration failures, experienced higher temporal demand and frustration. A recommendation that may follow from the above workload–accuracy correlations is that researchers who use eye-tracking may wish to consider measures to reduce workload in order to improve eye-tracking accuracy. For example, researchers should use concise instructions and provide adequate breaks.

Eye-tracker calibration verification in our study left some room for improvement. Although the calibration was rigorous, the experimenter was still responsible for subjectively verifying the accuracy of the calibration. It should also be noted that the trials in our experiment did not feature major disturbances such as wind, sunlight, or vibrations. In trials that may involve repeated facial movements or disturbances, or repositioning of the eye-tracker glasses, accuracy is likely to worsen. That said, compared to other eye-trackers, the Tobii glasses have been found to be relatively resistant to accuracy degradation caused by slippage (Niehorster et al., 2020).

It is worth noting that in our experiment, the chinrest was used without a forehead attachment, as the participants’ faces, when wearing the eye-tracker, could not be accommodated within the setup otherwise. Evidence from the scene camera indicated that participants’ head movements were minimal during the chinrest trials (see Fig. 5 for an example of one trial, which demonstrates limited movement of the dots, and Table 4 for numerical results). Consequently, it can be reasonably inferred that the findings of this study would not deviate significantly if the participants’ heads were fully restrained.

Another limitation of the current study is that it only examined angular distances from a target in a number of standard tasks. Future research could examine the capabilities of mobile eye-trackers for a more comprehensive set of measures (see Ehinger et al., 2019, who developed a test battery consisting of 10 tasks to evaluate the Pupil Labs glasses eye-tracker).

## Conclusions

This study showed that the Tobii Pro Glasses 3 yield better eye-tracking accuracy than the Tobii Pro Glasses 2 during walking. Furthermore, for the Tobii 2, not restraining the head yielded better eye-tracking accuracy than when a chinrest was used. Finally, participants who experienced higher workload exhibited poorer eye-tracking accuracy, which suggests that the observed eye-tracking accuracy is a function not only of the eye-tracker itself but also of the state of the wearer. Future research could investigate the relative performance of this and other eye-trackers under a wider range of task conditions and participant samples.

## Data Availability

Data and scripts are available at 10.4121/442018c6-30eb-4439-a452-c0046726905c.

## Appendix 1: Results for Eye-Tracker Precision

The precision of the gaze for a participant in an experimental condition was calculated by computing the standard deviation of the angular distance θ over the time interval (10 seconds), and subsequently averaging the intervals of that participant and condition. Table 7 shows the results of precision.

According to a two-way repeated-measures ANOVA, there was a significant effect of the Tobii model, *F*(1,32) = 10.3, *p* = 0.003, partial η^2^ = 0.24, as well as of the level of dynamicity, *F*(2,64) = 6.42, *p* = 0.003, partial η^2^ = 0.17, but there was no significant Tobii model × dynamicity interaction, *F*(2,64) = 1.82, *p* = 0.171, partial η^2^ = 0.05.

**Table 7:** Precision per experimental condition (in degrees). The mean, standard deviation (*SD*), and median across participants are reported

Dynamicity condition	Eye-tracker	Mean	*SD*	Median	*n*
Seated, with chinrest	Tobii 2	1.22	0.77	1.00	36
Seated, without chinrest	Tobii 2	0.89	0.52	0.81	36
Walking	Tobii 2	1.29	1.01	0.92	36
Seated, with chinrest	Tobii 3	0.81	0.72	0.58	34
Seated, without chinrest	Tobii 3	0.77	0.82	0.45	33
Walking	Tobii 3	0.97	0.90	0.68	34

## Appendix 2: Results for Eye-Tracker Bias

The bias of the gaze for a participant in an experimental condition was calculated by computing the mean gaze point and target point in pixels over the time interval (10 seconds), computing the angular distance of those means using Eq. 1, and subsequently averaging the intervals of that participant and condition.

Table 8 shows the results of bias. According to a two-way repeated-measures ANOVA, there was a significant effect of the Tobii model, *F*(1,32) = 32.0, *p* < 0.001, partial η^2^ = 0.50, a significant effect of dynamicity, *F*(2, 64) = 4.60, *p* = 0.014, partial η^2^ = 0.13. Moreover, there was a significant Tobii model × dynamicity interaction, *F*(2, 64) = 5.53, *p* = 0.006, partial η^2^ = 0.15.

**Table 8:** Bias per experimental condition (in degrees). The mean, standard deviation (*SD*), and median across participants are reported

Dynamicity condition	Eye-tracker	Mean	*SD*	Median	*n*
Seated, with chinrest	Tobii 2	2.22	1.10	1.97	36
Seated, without chinrest	Tobii 2	1.60	1.26	1.13	36
Walking	Tobii 2	2.86	2.01	2.52	36
Seated, with chinrest	Tobii 3	1.52	0.90	1.35	34
Seated, without chinrest	Tobii 3	1.30	0.71	1.10	33
Walking	Tobii 3	1.37	0.85	1.26	34
